# Defining global priorities in hematopoietic transplantation and cellular therapy: a statement paper from EBMT Global Committee

**DOI:** 10.1038/s41409-026-03008-7

**Published:** 2026-08-11

**Authors:** Arnon Nagler, Norbert Claude Gorin, Christian Chabannon, Guillermo Puigventós, Romée Heemskerk, Isabel Sánchez-Ortega, Tuula Rintala, Yann Mahé, Emanuele Angelucci, Silvia Montoto, Michelle Kenyon, Hilda Mekelenkamp, Donal P. McLornan, Antonio M. Risitano, Mette Hazenberg, Anna Sureda, Ibrahim Yakoub Agha, Annalisa Ruggeri, Fabio Ciceri, Raffaella Greco

**Affiliations:** 1 Division of Hematology, Sheba Medical Center, Tel Hashomer, Israel; 2 Service d’Hématologie clinique et Thérapie cellulaire, Hôpital Saint-Antoine, APHP, Paris, France; 3 EBMT Paris study office; Department of Haematology, Saint Antoine Hospital; INSERM UMR 938, Sorbonne University, Paris, France; 4 Centre de Thérapie Cellulaire, Département de Biologie du Cancer, Institut Paoli-Calmettes Comprehensive Cancer Centre, Inserm-Aix-Marseille Université-AP-HM-IPC, CBT-1409 Marseille, France; 5 Community Relations, Marketing & Communications, EBMT Executive Office, Barcelona, Spain; 6 EBMT Chief Medical Officer, Executive Office, Barcelona, Spain; 7 Advocacy and Quality of Care, EBMT Executive Office, Barcelona, Spain; 8 EBMT Executive Office, Barcelona, Spain; 9 Hematology and Cellular Therapy Unit. IRCCS Azienda Ospedaliera Metropolitana. Plesso Ospedale Policlinico San Martino, Genova, Italy; 10 Barts Health NHS Trust, London, UK; 11 Department of Haematology, King’s College Hospital NHS Foundation Trust, London, UK; 12 Willem-Alexander Children’s Hospital, Department of Paediatrics, Leiden University Medical Center, Leiden, the Netherlands; 13 Department of Haematology, University College London Hospitals, London, UK; 14 University College London, London, UK; 15 Hematology and Hematopoietic Transplant Unit, Azienda Ospedaliera di Rilievo Nazionale San Giuseppe Moscati (A.O.R.N. Giuseppe Moscati), Avellino, Italy; 16 Department of Hematology, Amsterdam UMC, University of Amsterdam, Amsterdam, The Netherlands; 17 Clinical Hematology Department, Institut Català d’Oncologia-Hospitalet, Institut d’Investigació Biomèdica de Bellvitge (IDIBELL), University of Barcelona, Barcelona, Spain; 18 Centre Hospitalier Universitaire de Lille, University of Lille, INSERM U1286, Infinite, Lille, 59000 France; 19 IRCCS Ospedale San Raffaele, Vita-Salute San Raffaele University, Haematology and Bone Marrow Transplantation, Milano, Italy

**Keywords:** Stem-cell research, Haematological diseases

## Abstract

Hematopoietic cell transplantation, cellular and gene therapies, and (CAR)-T cells have revolutionized outcomes in hematologic disorders. However, major disparities persist in their availability, accessibility, and affordability, particularly in low- and middle-income countries (LMICs). The EBMT Global Committee aims to address these inequalities. A structured survey was distributed to EBMT members to identify regional and thematic priorities, assess current infrastructure, and assess willingness to contribute to global initiatives. Responses were analyzed descriptively and served as the basis for the current statement paper. A total of 145 responses from 43 countries were collected. Most respondents (67.1%) reported working in JACIE- or FACT-accredited centers. The highest priority regions for EBMT outreach were Eastern Europe (non-EU) (43.9%), Latin America (38.2%), and Africa (33.5%), followed by the Middle East (29.5%). The leading thematic priorities were education and training (74.3%), scientific collaboration (56.0%), and registry development (43.4%). Most respondents expressed willingness to actively contribute, particularly in educational activities (59.7%). Qualitative responses emphasized the need for practical guidelines, mentorship programs, telemedicine, virtual discussion/clinical rounds on difficult cases, and support for LMIC capacity building. The EBMT community strongly supports an expanded global role focused on education, capacity building, and collaboration in underserved regions.

## Introduction

Hematopoietic cell transplantation (HCT) and cellular immunotherapies, including chimeric antigen receptor T-cell (CAR-T) therapy and gene therapies, represent major advances in the treatment of hematologic malignancies and selected non-malignant disorders [1–4]. Despite their curative potential, access to these therapies remains highly uneven worldwide, with profound disparities between high-income countries and low- and middle-income countries (LMICs) [5–13]. Global reports from the Worldwide Network for Blood and Marrow Transplantation (WBMT), World Marrow Donor Association (WMDA), World health organization (WHO), European Society for blood and marrow transplantation (EBMT), Centre for International Blood and Marrow Transplant Research (CIBMTR) and others have consistently demonstrated that transplant activity is concentrated in a limited number of regions, leaving large populations underserved [7, 8, 14–16]. The challenges extend beyond infrastructure and include workforce limitations, lack of donor registries, inadequate supportive care systems, and financial constraints. These barriers are further amplified for cellular therapies such as CAR-T, which require complex manufacturing, advanced regulatory frameworks, and substantial financial resources. As a result, access to these innovative treatments remains extremely limited in many parts of the world. In the 2018 WBMT global survey, 93,105 HCT procedures were reported worldwide, including 48,680 autologous and 44,425 allogeneic transplants. Activity was highest in Europe, North America, East/Southeast Asia, and the Western Pacific, while Africa, parts of Latin America, South Asia, and several Eastern Mediterranean regions remained underserved, with some improvements in more recent years [17, 18]. Cost-adapted transplant strategies, like the use of fresh non-cryopreserved autologous grafts, but mainly recent innovative developments and especially haploidentical transplantation with post-transplant cyclophosphamide (PTCy), helped in part to narrow the geographic regional gaps and improve the availability of HCT in the LMIC [7, 15–20]. Nevertheless, substantial challenges remain, and considerable efforts are still required to further reduce these inequities. The EBMT Global Committee (EGC) was established in 2017 with an aim to address these challenges. The committee strives to extend the scope of haematopoietic cell transplantation and cellular therapy to transplant teams outside Europe, to share knowledge, medical training, scientific collaboration, and research experience, by promoting knowledge exchange, capacity building, and collaborative research beyond Europe, extending the reach of EBMT’s mission worldwide. To better define its strategic priorities and align its activities with the needs of the global transplant community, a survey was conducted among EBMT members to collect views on priority regions and thematic areas for the Committee. This study reports the results of that survey that served as the foundation for the new structure of the EGC (Global Committee | EBMT) and outlines its implications for the future role of the EGC as described in our current statement.

## Methods

A cross-sectional, web-based survey was conducted by the EBMT to identify global priorities, assess current practices, and evaluate member engagement in EBMT activities related to hematopoietic cell transplantation (HCT) and cellular therapies. The survey methodology was designed in accordance with previously published EBMT survey approaches [21].

The questionnaire (supplementary Appendix 1) was developed by the EGC and included both closed-ended and open-ended questions, covering domains such as institutional characteristics (including accreditation status), geographic priorities for outreach, thematic areas of interest (education, research, registry participation, accreditation), and willingness of EBMT members to contribute to global initiatives. Free-text fields were included to capture qualitative insights and suggestions.

The survey was distributed electronically to EBMT members via official EBMT communication channels, including email mailing lists and society platforms, and was open from 6 May 2025 until 7 July 2025.

The study was approved by the EBMT Board of Directors. All methods were performed in accordance with the relevant guidelines and regulations, including the principles of the Declaration of Helsinki. Participation in the survey by EBMT transplant center representatives was voluntary. No patient-level data were collected as part of the survey. In general, patient data included in EBMT registry studies are submitted by participating transplant centers following written informed consent obtained in accordance with local ethical and regulatory requirements. The studies are approved by the relevant EBMT working party, the Chaim Sheba Medical Center Helsinki Committee (SMC-770-920), and the Israeli Ministry of Health. The study was conducted under the guidance of the EBMT and is reported in accordance with the STROBE recommendations.

## Results

**Fig. 1:**
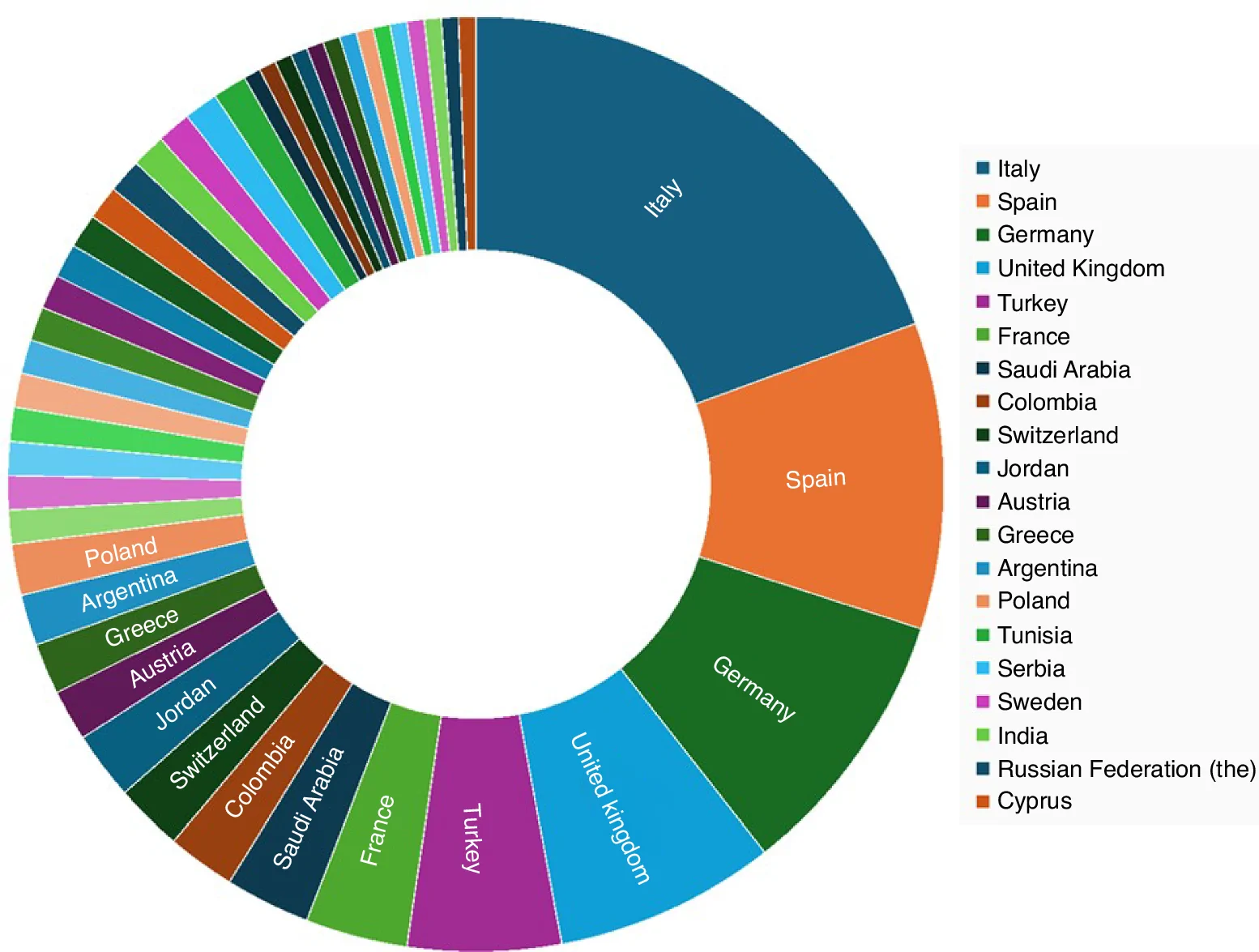
Respondent distribution by country. Distribution of survey responses by country, with the highest contributions from Italy, Spain, Germany, the United Kingdom, Turkey, and France.

**Fig. 2:**
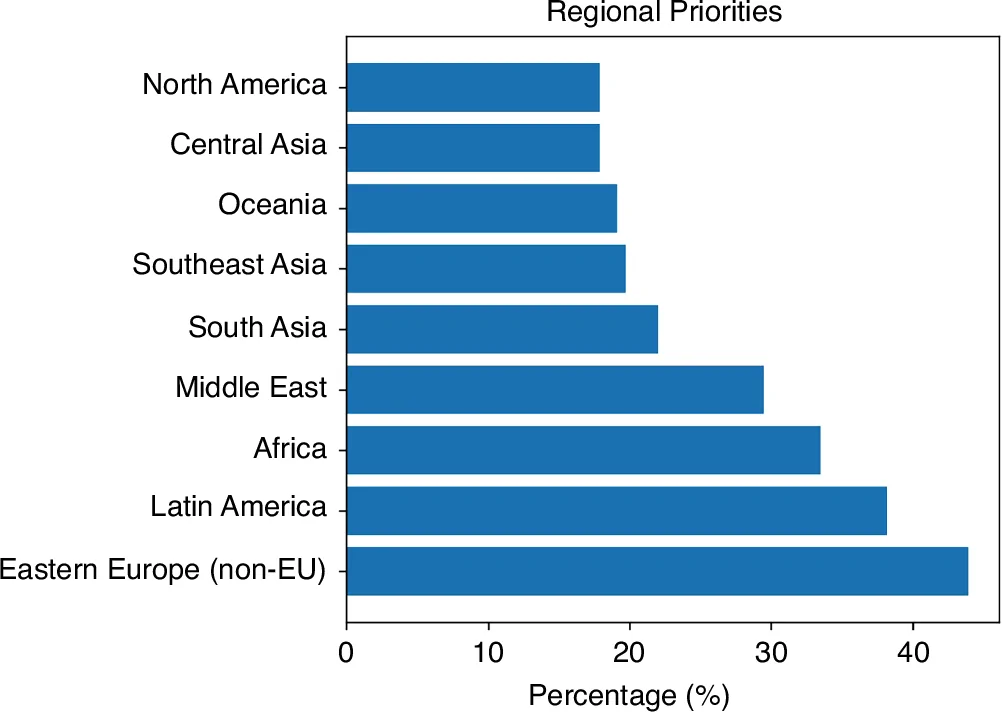
Regional priorities for EBMT Global Committee outreach. Regions prioritized by respondents for EBMT Global Committee outreach and collaboration.

The survey was distributed to 730 EBMT centers. A total of 145 centers from 43 countries responded to the survey (supplementary Appendix 2). Responses were predominantly from Europe, with the highest representation from Italy (≈20%), Spain (≈18%), Germany (≈15%), the United Kingdom (≈12%), Turkey (≈6–7%), and France (≈5%) (Fig. 1). Most respondents (67.1%) reported working in JACIE- or FACT-accredited centers (Table 1). The highest priority regions for EBMT outreach were Eastern Europe (non-EU) (43.9%), Latin America (38.2%), and Africa (33.5%), followed by the Middle East (29.5%) (Fig. 2; Table 2). Approximately 22% of the participating centers identified South Asia as the highest -priority region for EBMT outreach, while 19.7% identified Southeast Asia as the highest-priority region (Table 2).

**Table 1:** JACIE or FACT Accreditation.

Answer Choices	Responses (%) Centers No	
Yes	67.05	118
No	32.95	58

*No* number, *JACIE* Joint Accreditation Committee of the International Society for Cellular Therapy (ISCT) and the European Society for Blood and Marrow Transplantation (EBMT), *FACT* Foundation for the Accreditation of Cellular Therapies.

**Table 2:** Geographical areas identified as priorities for EBMT global committee outreach by survey respondents.

Answer Choices	Responses (%)	No of centers
Africa	33.53	58
Middle East	29.48	51
South Asia (e.g. India, Pakistan, Sri Lanka)	21.97	38
Southeast Asia (e.g. Vietnam, Thailand, Philippines, Malaysia)	19.65	34
Central Asia (e.g. Kazakhstan, Uzbekistan)	17.92	31
Latin America (e.g. Argentina, Brazil, Mexico)	38.15	66
Eastern Europe (non-EU)	43.93	76
Oceania (e.g. Australia, New Zealand)	19.08	33
North America	17.92	31
Other (please specify)	6.94	12

*No* number, *EU* Europe.

**Fig. 3:**
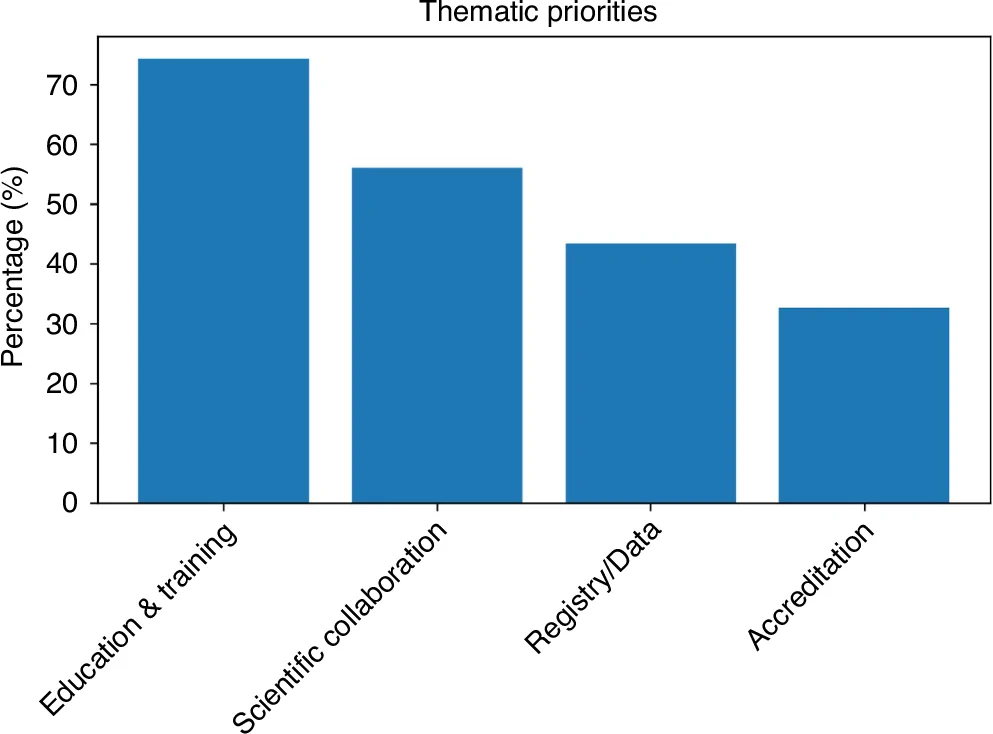
Thematic priorities of the Global Committee. Key thematic areas identified by respondents for Global Committee activities.

**Fig. 4:**
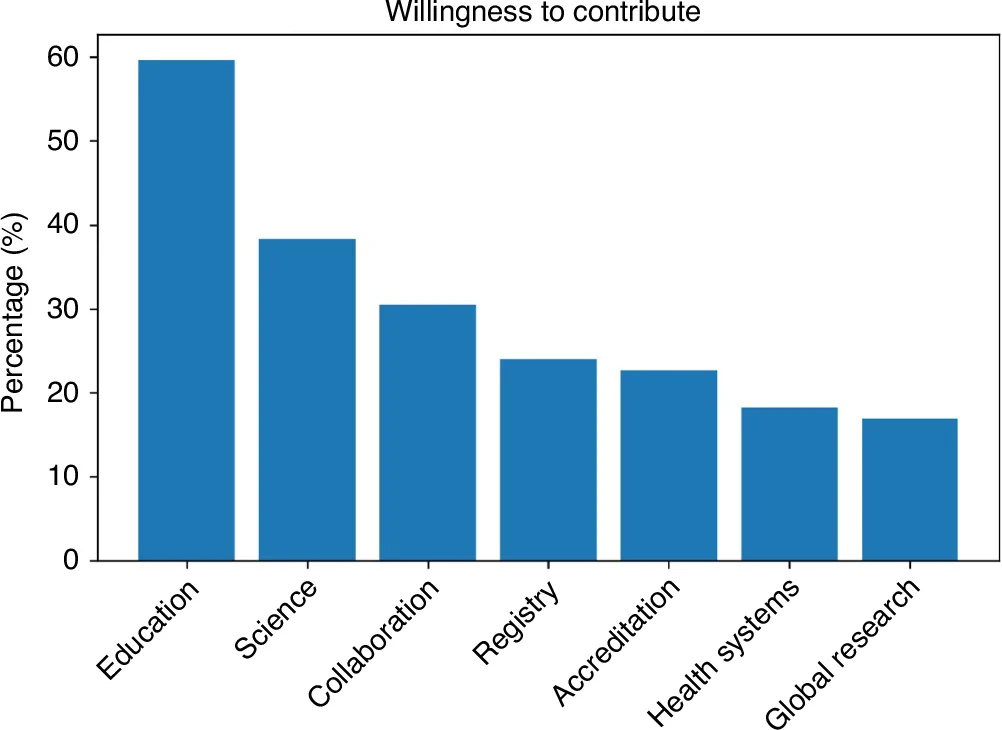
Willingness of members to contribute. Areas in which respondents are willing to actively contribute to Global Committee initiatives.

The leading thematic priority was education and training (74.3%), followed by scientific collaboration (56.0%), registry development (43.4%), and accreditation support (32.6%) (Fig. 3). Finally, most respondents expressed willingness to actively contribute, particularly in educational activities (59.7%), scientific work (38.3%), registry activities (24.0%), collaboration with external societies (30.5%), and accreditation support (22.7%) (Fig. 4).

Free-text responses highlighted several recurring themes: development of practical guidelines for establishing transplant programs in LMICs, expansion of education, training, and mentorship programs, increased use of telemedicine and virtual learning platforms, creation of structured international collaborations and partnerships, need for inclusive, multidisciplinary education, including nurses and allied professionals, as well as support for local adaptation of guidelines and clinical practices. Specific emphasis was given by the survey responders to education and training with multiple suggestions emphasizing education and training as central priorities for the EGC, including expansion of structured programs such as the EBMT curriculum, examination pathways, and international fellowships, alongside support for developing transplant units in LMICs.

Respondents highlighted the need to adapt existing tools and guidelines to local healthcare settings, incorporate hands-on training, and ensure applicability across diverse patient populations and diseases. There was also a strong call to broaden education beyond physicians to include multidisciplinary teams, particularly to facilitate the adoption of cellular therapies. In addition, participants advocated for the development of structured inter-regional collaboration platforms such as joint webinars, virtual discussion/clinical rounds on difficult cases, and mentorship programs, as well as increased use of telemedicine and virtual learning to enhance accessibility while minimizing costs.

Respondents from under-resourced regions underscored the need for regulatory guidance and capacity building to support the establishment of internationally competitive transplant programs. Respondents emphasized the importance of practical, capacity-building measures, including sharing data and expertise, providing free educational activities, and supporting data entry and management. Key priorities also include delivering training and clinical support, developing educational programs and fellowships, and offering consultation to emerging centers. Finally, participants highlighted the need for implementation of guidelines/recommendations, dissemination of best practices, and strengthening of quality assurance frameworks to support sustainable program development.

As for suggestions for the future, respondents proposed several strategic directions for the future role of the EGC, emphasizing the need for practical, stepwise guidelines to support the establishment of transplant units in LMICs, including clearly defined minimal requirements for infrastructure, laboratory, and clinical capacity. Participants highlighted the importance of strengthening international collaboration through joint scientific meetings, continuing medical education initiatives, and inclusive partnerships across regions. There was also a strong call to enhance the Committee’s role as a global bridge by fostering collaborative research tailored to local needs and improving representation from under-resourced regions. Additional priorities included supporting the diagnosis and management of hematologic diseases in developing countries, establishing structured mentorship programs linking experienced and emerging centers, and expanding educational resources particularly through multilingual content and training for non-physician staff.

## Discussion

The current statement paper, prepared on behalf of the EGC, is based on a structured survey distributed to EBMT centers to identify regional and thematic priorities and to assess the willingness of centers to contribute to future EGC initiatives. The survey also served as the foundation for the recently established structure of the EGC.

EBMT has extensive experience in transplant activity surveys, registry-based research, quality standards, and accreditation. Building on this expertise, the present survey was designed to identify priorities for reducing persistent disparities in the availability, accessibility, and affordability of hematopoietic cell transplantation, cellular therapies, and emerging immunotherapies, particularly in LMICs, through education, collaboration, and capacity building.

Rather than simply describing current practice, the survey provides a strategic framework for the future activities of the EGC. The findings reinforce previous reports identifying education and workforce development as major barriers to the expansion of transplantation and cellular therapy in LMICs and are consistent with international initiatives emphasizing capacity building as a prerequisite for improving cancer care in resource-limited settings.

A key strength of the survey is the clear alignment between identified needs and actionable priorities. Education and training emerged as the dominant theme, reinforcing previous observations that workforce development is a critical limiting factor in LMICs [14, 16, 17]. This is consistent with global oncology frameworks, which emphasize capacity building as a cornerstone for improving cancer outcomes in resource-limited settings [9, 20, 22].

Likewise, the prioritization of Eastern Europe (non-EU), Latin America, Africa, and South and Southeast Asia is consistent with regions where transplant activity continues to expand despite important challenges related to infrastructure, workforce, and access to advanced therapies [7, 8, 17].

An important observation emerging from the survey is the strong willingness of EBMT members to actively participate in educational, mentoring, and collaborative initiatives. This highlights the opportunity for the EGC to leverage the expertise of its membership to support sustainable global development. At the same time, the findings reinforce the growing recognition that scientific innovation must be accompanied by strategies that promote equitable access. While highly specialized therapies such as CAR-T cells represent major therapeutic advances, their current cost and complexity risk widening global disparities [11, 12]. Simplified and cost-adapted transplant approaches, such as fresh non-cryopreserved autologous grafts and haploidentical transplantation, and simplified treatment platforms may offer more scalable solutions in LMICs [7, 19, 20].

The survey findings also informed the recent reorganization of the EGC around regional leadership and thematic priorities. The Committee’s future activities are expected to focus on education and training, capacity building, registry development, quality improvement, and international scientific collaboration. These priorities reflect the interpretation of the survey findings by the authors and the EGC and are intended to guide future strategic development rather than represent direct outcomes of the survey. Their implementation is expected to be progressive, beginning with initiatives that can be established in the short term, such as educational programs, mentoring activities, and collaborative networks, while longer-term objectives, including strengthening registries, promoting quality standards and accreditation, and supporting sustainable transplant and cellular therapy programs in LMICs, will require continued collaboration with regional partners and international organizations.

Several limitations should be acknowledged. As with all survey-based studies, the findings are subject to self-reporting and potential selection bias. In addition, only ~20% of EBMT centers participated, with responses predominantly originating from European countries and no participation from centers in China. Consequently, the perspectives of several important LMIC regions may be underrepresented, and the analysis remains primarily descriptive. Nevertheless, the survey provides valuable insights into the priorities and perspectives of a broad international transplant community and offers an important foundation for future global initiatives.

In conclusion, the EGC represents an important strategic extension of EBMT’s mission to promote equitable access to transplantation and cellular therapies worldwide. The survey demonstrates broad support within the EBMT community for expanding international collaboration, education, and capacity building, particularly in LMICs. Importantly, the findings have already informed the new organizational structure of the EGC and will continue to guide its future strategic priorities and implementation activities.

## Supplementary information

Supplementary appendix - centers

Supplemental material - survey questionary
