# Publisher Correction: defining global priorities in hematopoietic transplantation and cellular therapy: a statement paper from the EBMT global committee

**DOI:** 10.1038/s41409-026-03046-1

**Published:** 2026-10-12

**Authors:** Arnon Nagler, Norbert Claude Gorin, Christian Chabannon, Guillermo Puigventós, Romée Heemskerk, Isabel Sánchez-Ortega, Tuula Rintala, Yann Mahé, Emanuele Angelucci, Silvia Montoto, Michelle Kenyon, Hilda Mekelenkamp, Donal P. McLornan, Antonio M. Risitano, Mette Hazenberg, Anna Sureda, Ibrahim Yakoub Agha, Annalisa Ruggeri, Fabio Ciceri, Raffaella Greco

**Affiliations:** 1 Division of Hematology, Sheba Medical Center, Ramat Gan, Israel; 2 Service d’Hématologie Clinique et Thérapie Cellulaire, Hôpital Saint-Antoine, APHP, Paris, France; 3 EBMT Paris study office; Department of Haematology, Saint Antoine Hospital; INSERM UMR 938, Sorbonne University, Paris, France; 4 Centre de Thérapie Cellulaire, Département de Biologie du Cancer, Institut Paoli-Calmettes Comprehensive Cancer Centre, Inserm-Aix-Marseille Université-AP-HM-IPC, Marseille, France; 5 Community Relations, Marketing & Communications, EBMT Executive Office, Barcelona, Spain; 6 EBMT Chief Medical Officer, Executive Office, Barcelona, Spain; 7 Advocacy and Quality of Care, EBMT Executive Office, Barcelona, Spain; 8 EBMT Executive Office, Barcelona, Spain; 9 Hematology and Cellular Therapy Unit. IRCCS Azienda Ospedaliera Metropolitana. Plesso Ospedale Policlinico San Martino, Genova, Italy; 10 Barts Health NHS Trust, London, UK; 11 Department of Haematology, King’s College Hospital NHS Foundation Trust, London, UK; 12 Willem-Alexander Children’s Hospital, Department of Paediatrics, Leiden University Medical Center, Leiden, the Netherlands; 13 Department of Haematology, University College London Hospitals, London, UK; 14 University College London, London, UK; 15 Hematology and Hematopoietic Transplant Unit, Azienda Ospedaliera di Rilievo Nazionale San Giuseppe Moscati (A.O.R.N. Giuseppe Moscati), Avellino, Italy; 16 Department of Hematology, Amsterdam UMC, University of Amsterdam, Amsterdam, The Netherlands; 17 Clinical Hematology Department, Institut Català d’Oncologia-Hospitalet, Institut d’Investigació Biomèdica de Bellvitge (IDIBELL), University of Barcelona, Barcelona, Spain; 18 Centre Hospitalier Universitaire de Lille, University of Lille, Infinite, INSERM, Lille, France; 19 IRCCS Ospedale San Raffaele, Vita-Salute San Raffaele University, Haematology and Bone Marrow Transplantation, Milano, Italy

**Keywords:** Stem-cell research, Haematological diseases

Correction to: *Bone Marrow Transplantation* https://doi.org/10.1038/s41409-026-03008-7, published online 11 August 2026

In this article, in the 4th paragraph of the Method section, additional words have been added during the correction process: “are approved by the relevaThe study was approved by the EBMT Board of Directors. All methods were performed in accordance with the relevant ...”

The sentence should be read: “The study was approved by the EBMT Board of Directors. All methods were performed in accordance with the relevant ...”

The original article has been corrected.

