# Supplementary material for: Defining global priorities in hematopoietic transplantation and cellular therapy: a statement paper from EBMT Global Committee
Source: Bone Marrow Transplant. 2026 Aug 11;61(11):1475–9. doi: 10.1038/s41409-026-03008-7 (PMC13743486; doi:10.1038/s41409-026-03008-7)
Supplement: Supplementary file 1 [file 41409_2026_3008_MOESM2_ESM.pdf]

EBMT Global Committee Stakeholder Survey

1.

The EBMT Global Committee is committed to fostering scientific collaboration and engagement beyond Europe, extending the reach of EBMT's mission worldwide. This brief survey aims to collect your views on priority regions and thematic areas for the Committee, and to identify those interested in contributing to its future activities.

We would be grateful for your insights. The survey should take no more than 4 minutes to complete.
All responses will remain confidential unless you indicate otherwise.

SECTION 1: Contact Information - Please complete the following details regarding your EBMT centre:

1. Full name:

[ ]

2. CIC (Centre Identification Code):

[ ]

3. Email Address:

[ ]

4. JACIE or FACT Accreditation:

- ○ Yes
- ○ No

5. □ **SECTION 2:** Regional Priorities: Which geographical areas should the EBMT Global Committee prioritise in its outreach and collaboration efforts? (Please tick all that apply – a reference list of countries involved in EBMT25 is included below for guidance)

- □ Africa
- □ Middle East
- □ South Asia (e.g. India, Pakistan, Sri Lanka)
- □ Southeast Asia (e.g. Vietnam, Thailand, Philippines, Malaysia)
- □ Central Asia (e.g. Kazakhstan, Uzbekistan)
- □ Latin America (e.g. Argentina, Brazil, Mexico)
- □ Eastern Europe (non-EU)
- □ Oceania (e.g. Australia, New Zealand)
- □ North America
- □ Other (please specify)

[ ]

6. □ **SECTION 3 (a):** Thematic Priorities: Which key areas of activity should the Global Committee prioritise? (Please tick all that apply, and provide more detail where possible)

- ☐ Scientific collaboration
- ☐ Education and training
- ☐ Accreditation support
- ☐ Registry and data participation
- ☐ Other (please specify)

[ ]

7. **SECTION 3 (b):** Use this space to provide more detail

Specify Scientific collaboration	
Specify Education and training	
Specify Accreditation support	
Specify Registry and data participation	

8. □ **SECTION 4 (a):** Personal Involvement: Are you personally interested in contributing to the Global Committee in any of the following areas? (Please tick all that apply and specify your focus or expertise)

- ☐ Science
- ☐ Education
- ☐ Accreditation
- ☐ Registry
- ☐ Health system development / health economics
- ☐ Research on diseases more prevalent outside Europe
- ☐ Collaboration with external societies / registries
- ☐ Other (please specify)

[ ]

9. **SECTION 4 (b)**: Use this space to provide more detail

Science	
Education	
Accreditation	
Registry	
Health system development / health economics	
Research on diseases more prevalent outside Europe	
Collaboration with external societies / registries	

10. **SECTION 5**: Suggestions for the Future: Do you have any suggestions, proposals, or reflections to share regarding the future role and structure of the EBMT Global Committee?
