# Supplementary material for: Defining global priorities in hematopoietic transplantation and cellular therapy: a statement paper from EBMT Global Committee
Source: Bone Marrow Transplant. 2026 Aug 11;61(11):1475–9. doi: 10.1038/s41409-026-03008-7 (PMC13743486; doi:10.1038/s41409-026-03008-7)
Supplement: Supplementary file 2 [file 41409_2026_3008_MOESM1_ESM.docx]

**Supplementary Appendix: Contributing Centers**

| **Center CIC** | **Centre name** | **Country** |
| --- | --- | --- |
| 104 | Klinikum Chemnitz gGmbH | Germany |
| 125 | Dortmunder Centrum fur Zelltransplantation (DCZ) | Germany |
| 134 | Universitaet Bonn | Germany |
| 145 | Robert_Bosch_Krankenhaus | Germany |
| 160 | Hopital Necker Adults | France |
| 162 | Centro di Riferimento Oncologico | Italy |
| 179 | Universidade Federal do Rio de Janeiro | Brazil |
| 183 | Centre National de Greffe de Moelle | Tunisia |
| 189 | Klinikum Schwabing | Germany |
| 194 | Hopital de Meaux | France |
| 202 | University Hospital \| Basel | Switzerland |
| 203 | Leiden University Hospital | Netherlands (the) |
| 204 | Klinik fuer Innere Medzin III | Germany |
| 205 | Imperial College Hammersmith London | United Kingdom of Great Britain and Northern Ireland (the) |
| 209 | University Hospital Gasthuisberg | Belgium |
| 212 | Karolinska University Hospital | Sweden |
| 214 | Hospital Clinic | Spain |
| 217 | IRCCS Ospedale Policlinico San Martino | Italy |
| 224 | University College London Hospital | United Kingdom of Great Britain and Northern Ireland (the) |
| 227 | Medizinische Universitaet Wien | Austria |
| 230 | Programme de Transplantation&Therapie Cellulaire | France |
| 233 | CHU Besancon Franche-Comte | France |
| 236 | Hospital de la Princesa | Spain |
| 244 | Glasgow Royal Infirmary | United Kingdom of Great Britain and Northern Ireland (the) |
| 248 | Ospedale Civile | Italy |
| 254 | St James University Hospital Leeds | United Kingdom of Great Britain and Northern Ireland (the) |
| 256 | University Medical Center Schleswig-Holstein, Campus Kiel | Germany |
| 257 | Hope Directorate - St. James's Hospital | Ireland |
| 258 | Hadassah University Hospital | Israel |
| 260 | Hospital Santa Creu i Sant Pau | Spain |
| 271 | University Hospital Innsbruck | Austria |
| 274 | Institute G. Gaslini | Italy |
| 281 | Research Committee - University of Patras | Greece |
| 282 | Hospital Clinico de Valencia | Spain |
| 288 | Spedali Civili - Brescia | Italy |
| 290 | Klinikum Karlsruhe gGmbH | Germany |
| 291 | Inst. Portugues de Oncologia do Porto | Portugal |
| 300 | Inst. Portugues Oncologia | Portugal |
| 302 | University Hospital Center Rebro | Croatia |
| 304 | Azienda Ospedaliera Universitaria Careggi | Italy |
| 305 | Ospedale Infantile Regina Margherita | Italy |
| 306 | Unita Operativa di Ematologia | Italy |
| 307 | Universita Cattolica S. Cuore | Italy |
| 333 | Roosevelt Hospital | Slovakia |
| 336 | Charite-CVK, University Medicine Berlin | Germany |
| 341 | Azienda Ospedaliera di Rilievo Nazionale | Italy |
| 345 | Rambam Medical Center | Israel |
| 359 | Universitaetsklinium Magdeburg | Germany |
| 392 | U.O.D Trapianti di midollo osseo | Italy |
| 396 | Kuopio University Hospital | Finland |
| 397 | King Faisal Specialist Hospital & Research Centre | Saudi Arabia |
| 399 | Hacettepe University Children`s Hospital | Turkey |
| 410 | Hospital Univ.Materno Infantil Gregorio Maranon | Spain |
| 415 | Presidio Ospedaliero Ca`Foncello | Italy |
| 420 | EUROCORD | France |
| 421 | Hospital Alvaro Cunqueiro - Complejo Hospitalario Universitario de Vigo | Spain |
| 422 | Hospital Vall d'Hebron | Spain |
| 427 | Fundeni Clinical Institute Adults | Romania |
| 434 | Singapore General Hospital | Singapore |
| 444 | King Abdul - Aziz Medical City | Saudi Arabia |
| 451 | Unidad de trasplantes Clinica Leon XIII | Colombia |
| 456 | ALBERTS CELLULAR THERAPY | South Africa |
| 465 | BMJH - SANKALP | India |
| 467 | Banc de Sang i Teixits | Spain |
| 499 | Instituto de Prevision Social | Paraguay |
| 513 | Klinikum Grosshadern | Germany |
| 520 | Clinic of Hematology and Cellular Therapy | Russian Federation (the) |
| 521 | Royal Manchester Children's Hospital | United Kingdom of Great Britain and Northern Ireland (the) |
| 526 | IRCCS, Casa Sollievo della Sofferenza | Italy |
| 533 | Universitaetsklinikum Jena | Germany |
| 539 | St. George's Hospital London | United Kingdom of Great Britain and Northern Ireland (the) |
| 565 | University Hospital Maastricht | Netherlands (the) |
| 566 | Addenbrookes Hospital Cambridge | United Kingdom of Great Britain and Northern Ireland (the) |
| 574 | Olomouc University Hospital | Czechia |
| 579 | Welsh Bone Marrow Donor Registry | United Kingdom of Great Britain and Northern Ireland (the) |
| 580 | King Hussein Cancer Centre Adult BMT Program | Jordan |
| 582 | Clinic of Hematology | Serbia |
| 587 | Grande Ospedale Metropolitano Bianchi Melacrino Morelli - Centro Unico Trapianti A. Neri | Italy |
| 589 | Baskent University Hospital | Turkey |
| 598 | Hospital Universitario Donostia | Spain |
| 608 | Great Western Hospital | United Kingdom of Great Britain and Northern Ireland (the) |
| 613 | ICO-Hospital Universitari Germans Trias i Pujol | Spain |
| 620 | University of Ankara | Turkey |
| 623 | Azienda Ospedaliera Universitaria Integrata Verona | Italy |
| 628 | Ege University Medical School | Turkey |
| 629 | Armed Forces Bone Marrow Transplant Centre | Pakistan |
| 638 | Hospital Hirslanden | Switzerland |
| 648 | University of Debrecen Clinical Center | Hungary |
| 660 | Arcispedale S. Maria Nuova | Italy |
| 663 | University Hospital La Fe | Spain |
| 668 | Hospital Sant Joan de Deu Materno-Infantil | Spain |
| 676 | CHRU NANCY | France |
| 678 | Children`s University Hospital | Poland |
| 711 | The Children's Hospital at Westmead | Australia |
| 718 | Charles University Hospital | Czechia |
| 735 | Hospital Morales Meseguer | Spain |
| 737 | Clinica Universitaria de Navarra | Spain |
| 740 | University Hospital \| Linkoeping | Sweden |
| 742 | Fundacion Favaloro | Argentina |
| 749 | Klinikum Oldenburg | Germany |
| 751 | St. Savvas Oncology Hospital | Greece |
| 752 | St Sophia Children's Hospital | Greece |
| 756 | Tor Vergata University of Rome | Italy |
| 769 | Hospital Universitario Virgen del Rocio | Spain |
| 778 | Sheffield Royal Hallamshire | United Kingdom of Great Britain and Northern Ireland (the) |
| 787 | University Regensburg | Germany |
| 790 | Azienda Ospedaliero Universitaria | Italy |
| 792 | Ospedale Policlinico | Italy |
| 794 | Sezione di Ematologia | Italy |
| 795 | Azienda Ospedaliero Universitaria Pisana | Italy |
| 797 | S. Bortolo Hospital | Italy |
| 799 | University Clinical Centre in Gdansk | Poland |
| 809 | University Hospital Erlangen | Germany |
| 811 | Centro Trapianti Unico Di CSE Adulti e Pediatrico A. O Brotzu | Italy |
| 813 | IRCCS San Raffaele Hospital Scientific Institute | Italy |
| 820 | CHUV Centre Hospitalier Universitaire Vaudois | Switzerland |
| 848 | Aarhus University | Denmark |
| 858 | King Faisal Specialist Hospital and Research Center | Saudi Arabia |
| 859 | National Hospital of Haematological Diseases | Bulgaria |
| 891 | Hematology Center R.H Yeolyan | Armenia |
| 927 | ASST Valle Olona - Ospedale di Circolo di Busto Arsizio | Italy |
| 928 | Medical School University of Salerno | Italy |
| 943 | Koc University Hospital | Turkey |
| 981 | King Faisal Specialist Hospital and Research Centre | Saudi Arabia |
| 985 | FOSCAL Internacional of the FOSUNAB Foundation | Colombia |
| 993 | University Hospital of Oran | Algeria |
| 996 | Antwerp University Hospital (UZA) | Belgium |
| 998 | Instituto de Cancerologia S.A | Colombia |
| 1000 | Instituto de Transplante y Alta Complejidad | Argentina |
| 1001 | Bahrain Oncology Center | Bahrain |
| 1002 | FUNDALEU | Argentina |
| 1003 | Meyer University Children Hospital | Italy |
| 1004 | Afyonkarahisar Health Sciences University | Turkey |
| 1033 | University Hospital Split | Hungary |
| 1036 | Akbar Niazi Teaching Hospital | Pakistan |
| 1045 | Hospital Nove de Julho | Brazil |
| 1052 | Abu Dhabi Stem Cell Center | United Arab Emirates (the) |
| 1058 | King Hussein Cancer Centre Pediatric BMT Program | Jordan |
| 1078 | Hospital General Universitario Dr Balmis | Spain |
| 1079 | Memorial Sisli Hastanesi | Turkey |
| 1085 | University Hospital St. Marina | Bulgaria |
| 1089 | German Oncology Center | Cyprus |
| 1098 | Hospital San Vicente Fundacion | Colombia |
| 1099 | LIV HOSPITAL | Turkey |
| 1107 | Ukrainian National cancer institute | Ukraine |
